# A Para-Scrotal Pleomorphic Liposarcoma Mimicking As Another Scrotum (Pseudo-Scrotum): A Case Report

**DOI:** 10.7759/cureus.46569

**Published:** 2023-10-06

**Authors:** Kavita Jadhav, Rajalakshmi Venkateswaran, Ravi Landge, Akshay Kadam, Rihan Rashid

**Affiliations:** 1 General Surgery, Grant Government Medical College and Sir JJ Group of Hospitals, Mumbai, IND

**Keywords:** radiotherapy, radical surgery, pseudoscrotum, pleomorphic liposarcoma, soft tissue sarcoma

## Abstract

Liposarcomas are a subtype of soft tissue sarcomas arising from lipoblasts, a mesenchymal cell lineage that commonly arises from deep tissues of the body. Though these are the most common subtypes, their early diagnosis still remains a challenge due to their varied presentation as a soft benign appearing growth. The pleomorphic variant has complex management due to its high recurrence rate and resistance to chemoradiation. Scrotal liposarcomas have been reported. But in the present case, a 69-year-old male who presented with a pedunculated swelling in the left groin mimicking a left hemi-scrotal swelling. It was a left para-scrotal pleomorphic liposarcoma which was totally extra-scrotal, and not related to the spermatic cord or testes. So, this is a rare case with a review of the literature presented here.

## Introduction

Soft tissue sarcomas account for <1% of all solid malignancies of adulthood. The World Health Organization (WHO) has further classified these soft tissue sarcomas into 70 subtypes, of which liposarcoma is the most commonly detected subtype [1]. The WHO, in 2020 has further classified liposarcoma into 5 types: well-differentiated, dedifferentiated, myxoid, round cell, and pleomorphic liposarcoma [2]. Common sites of occurrence are the extremities (45-55%) and retroperitoneum (13-18%) [3].

In literature, intra-scrotal and para-testicular liposarcomas, spermatic cord liposarcomas have been reported. Para-scrotal which are totally extra-scrotal swellings or liposarcomas, not originating from the spermatic cord are rare.

In the present case, the patient developed deep perineal fat tissue liposarcoma, and the patient presented as left scrotal mass but not from the scrotum; it was para-scrotal appearing like the scrotum, and so labeled as pseudo-scrotum. It was a left para-scrotal low-grade pleomorphic liposarcoma.

## Case presentation

A 69-year-old male presented with complaints of a painless left scrotal swelling since 15 years which was progressively enlarging since 6 months of admission. On general examination, the vital parameters were within normal limits. 

**Figure 1:**
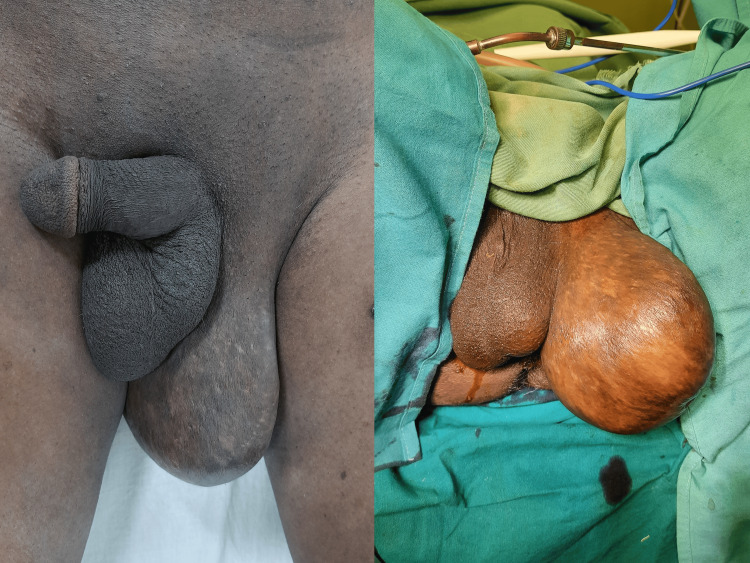
Left para-scrotal swelling appearing as pseudo-scrotum

Detailed physical examination revealed an enlarged and pedunculated left para-scrotal swelling with a soft to firm vague mass within. The left testis was displaced into the right hemi-scrotum, giving the appearance of scrotal swelling (Figure 1). Clinically the swelling was assumed to be a pedunculated lipomasarcoma considering history and examination findings.

**Figure 2:**
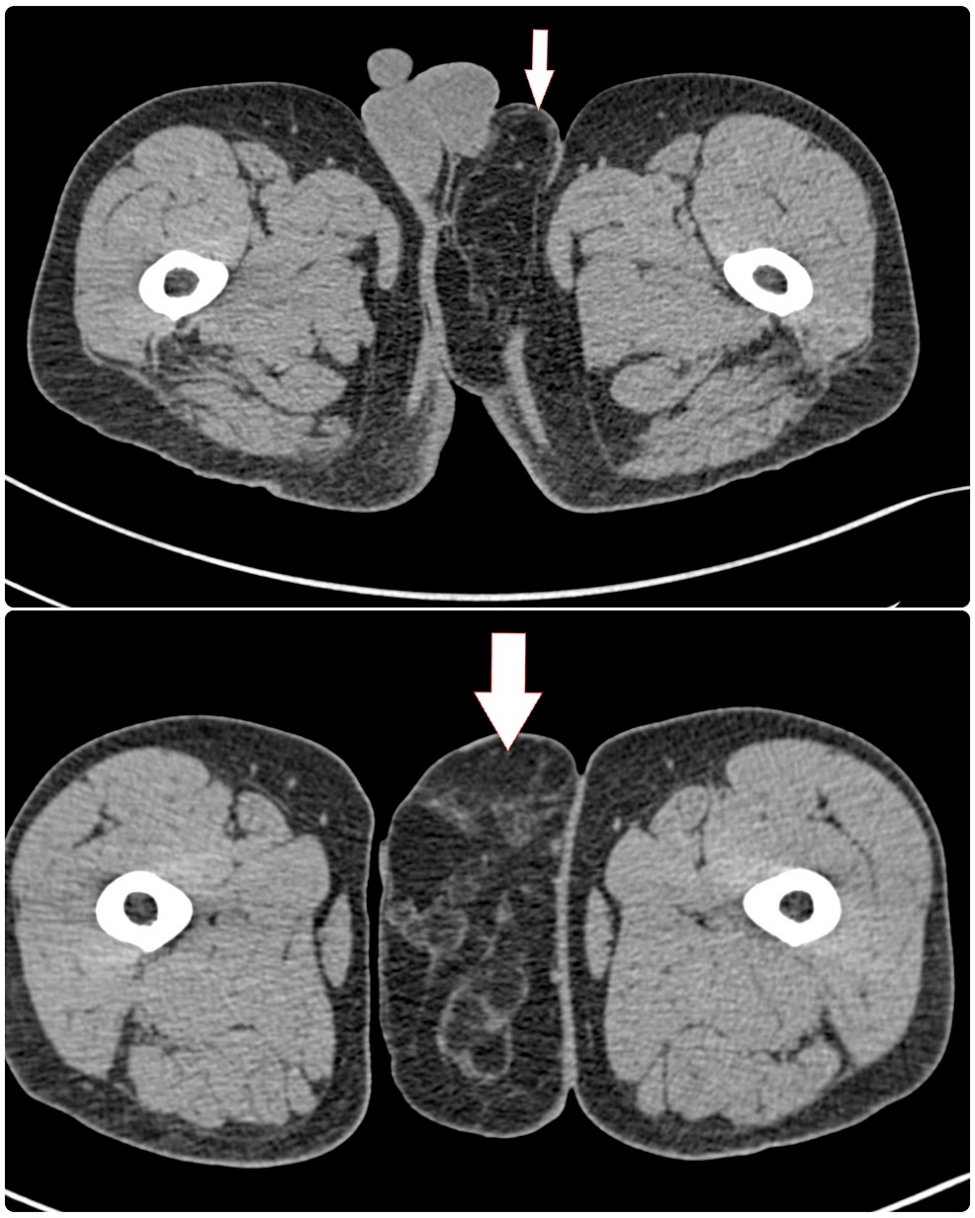
CECT images showing the origin of para-scrotal liposarcoma CECT: contrast-enhanced computed tomography

A contrast-enhanced computed tomography (CECT) scan of the pelvis was done which reported a pedunculated lesion with predominant fat density measuring 13.4 cm x 5.8 cm arising from the perineal region between the left scrotum and medial aspect of the left thigh. Its superior extent was broad-based extending into the whole anteroposterior perineum and was in continuity with the fat of the left inguinal, medial aspect of the gluteal region. Soft tissue densities and calcifications were noted (Figure 2). The left testis and spermatic cord were separate from the lesion but were deviated to the right side. The above findings were suspicious of malignant neoplasm of lipomatous in origin. 

**Figure 3:**
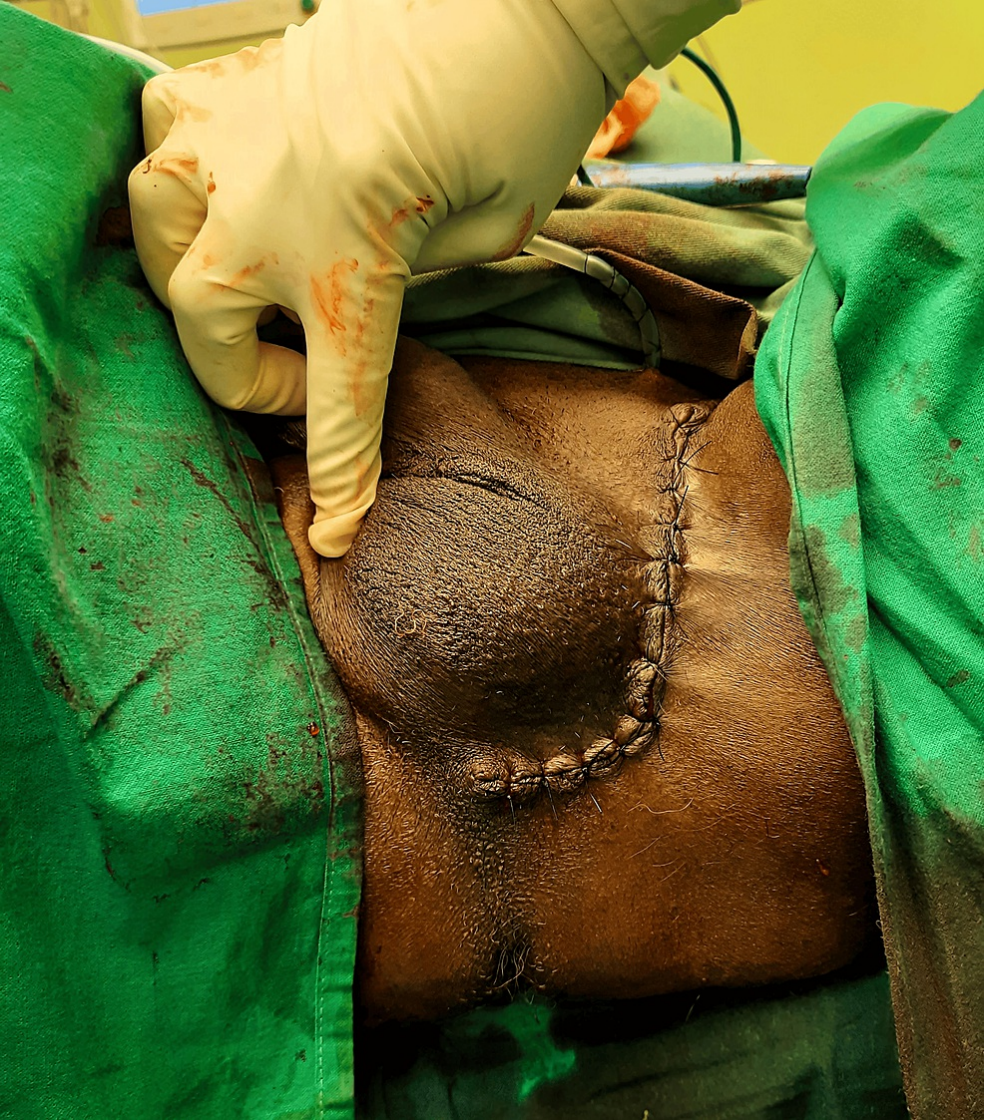
Postexcision suture site

Ultrasound-guided core biopsy was performed which was reported as normal adipose tissue cells with no atypia. The patient then underwent wide local excision of the tumor through an incision on the lateral aspect of the left hemi-scrotum (Figure 3).

**Figure 4:**
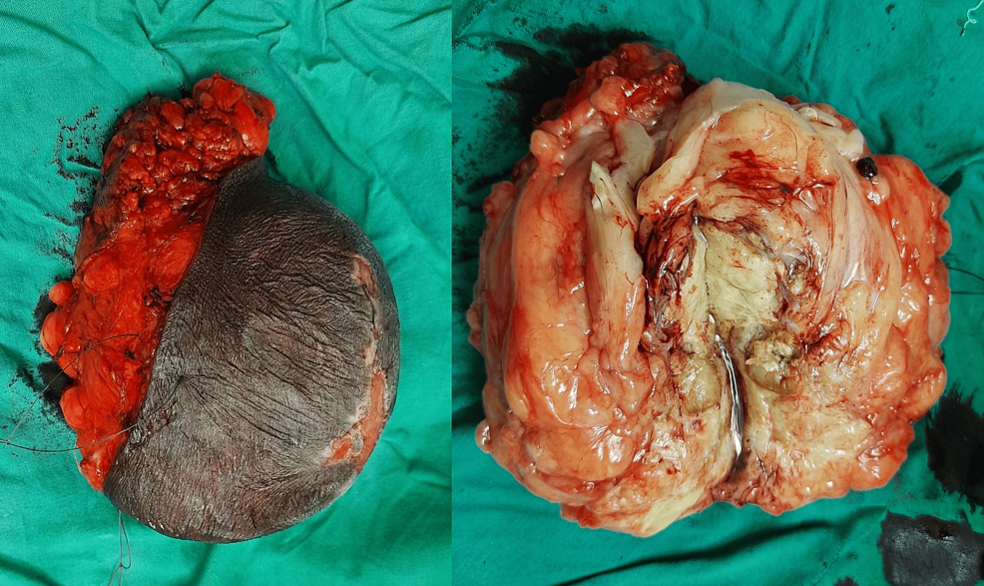
Excised specimen of para-scrotal liposarcoma and cut open specimen of liposarcoma

A gross examination of the tumor revealed a firm capsulated tumor with a yellowish-white firm necrotic center with the surrounding adipose tissue (Figure 4).

**Figure 5:**
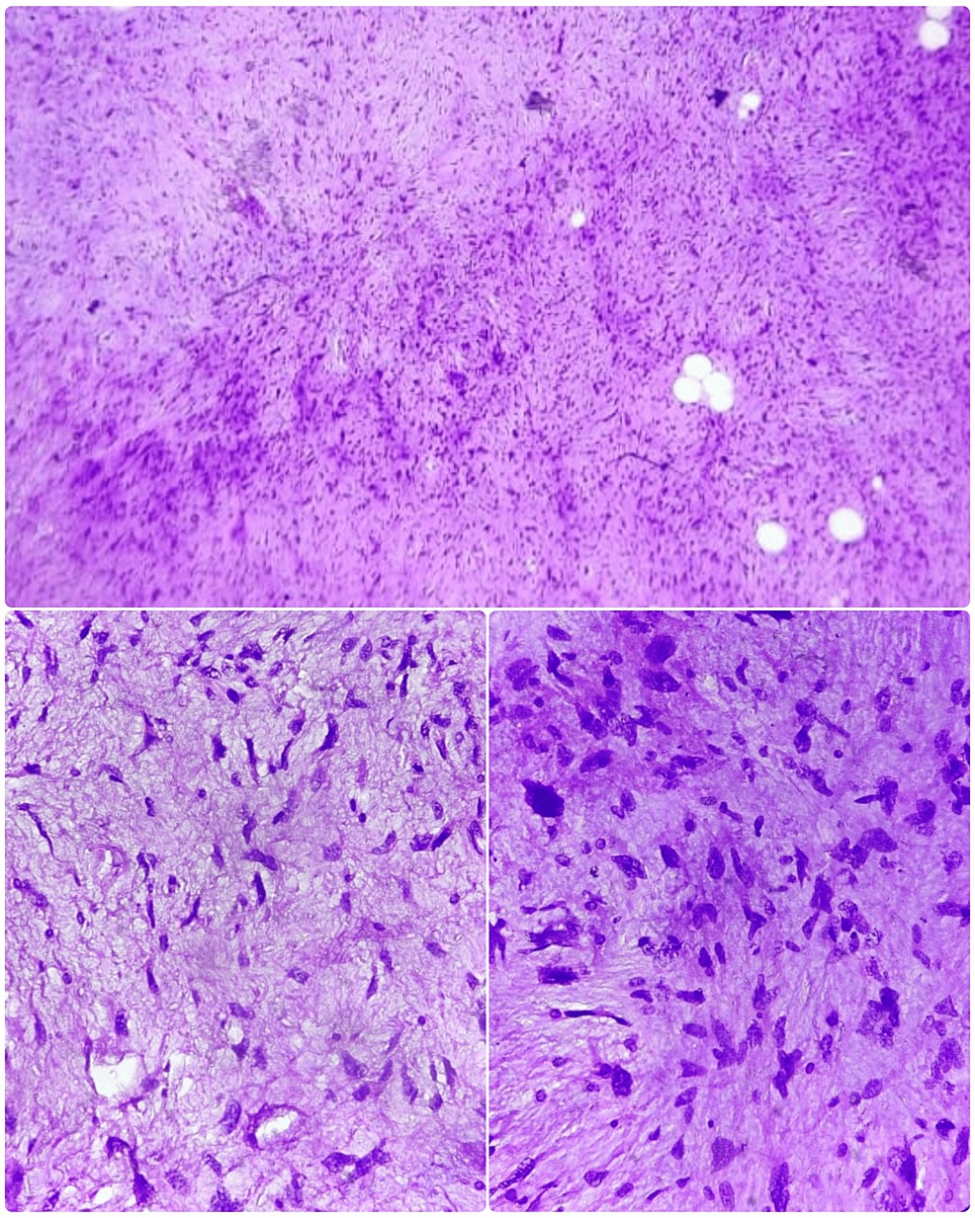
Histopathological image of pleomorphic liposarcoma

Histopathological analysis of the specimen showed R0 resection margins with low-grade pleomorphic lipoblasts and interspersed spindle cells, suggestive of a pleomorphic liposarcoma. The mitotic index was 4 mitosis/high power field, hence was reported to be a low-grade tumor (Figure 5). 

The post-operative period was uneventful. Post-procedure radiotherapy was given. A CECT pelvis was repeated at 6 months which showed no recurrence. Since then, the patient has been under regular follow-up and there has been no recurrence.

## Discussion

Lipoma is known as universal swelling as it can occur at anywhere in the body. Prolonged lipomas may become malignant leading to liposarcoma. Pleomorphic liposarcoma is the rarest variant of liposarcoma, accounting for <5% of all cases [4]. Its incidence has been found to be slightly higher in men as compared to women. The diagnosis of a liposarcoma clinically is often a challenge as they usually arise from deep soft tissues and become symptomatic at a very later stage once the tumor starts to cause compressive symptoms. In the index case, the dilemma in diagnosis occurred due to its varied presentation as a soft pedunculated growth which appeared as a pseudo-scrotal swelling.

In literature, intra-scrotal and peri-testicular or para-testicular (inside scrotum) liposarcomas have been reported. But para-scrotal which was outside the scrotum and bigger than the scrotum, low-grade pleomorphic liposarcoma has not been reported in the literature.

Accessory to clinical examination is CECT, magnetic resonance imaging (MRI), and biopsy of the lesion. The radiological investigations help in assessing the exact extent of the tumor, presence of calcifications, proximity of involvement of vital structures such as vessels, bowel, etc. On histopathological examination, these tumors show mononucleate/multinucleated giant cells, bizarre cells, and pleomorphic lipoblasts. Pleomorphic lipoblasts have hyperchromic nuclei, multiple nuclei, and univacuolated/multivacuolated cytoplasm [5].

These pleomorphic lipoblasts form only 10% of the tumor mass, hence a negative pre-operative biopsy report can often be misleading as happened with the present case [6]. Therefore, in a patient with radiological investigations pointing towards a lipomatous tumor, radical excision of the tumor should be planned, irrespective of the pre-operative biopsy report.

Wide local excision of superficial pleomorphic liposarcoma is the treatment of choice [5]. Ensuring negative margins is vital to prevent recurrence. In cases where vital structures such as the bowel and viscera are involved or infiltrated, radical excision of the tumor with adequate resection of vital structures might be required [4-6].

Adjuvant radiotherapy (RT) should be administered to manage micro-metastasis and in R1 resection cases. RT has been proven to increase the disease-free survival (DFS) period [7]. Wan et al. studied 555 patients of pleomorphic liposarcoma in 2021, wherein they showed that adjuvant RT was beneficial in tumors more than 10 cm and increased DFS [7]. Low-grade lesions do not require further targeted therapy. 

Old age, large tumors (>10 cm), presence of high mitotic index (>10 mitosis/hpf), positive surgical margins, and deep soft tissue sarcomas have been proven to be worse prognostic indicators [7]. Superficial tumors generally have a good prognosis due to their early presentation and feasibility of R0 resection. Low-grade superficial tumors usually do not require neoadjuvant chemoradiation. Regular strict surveillance should be done for a maximum period of 10 years [7]. 

In literature, many cases with para-testicular liposarcomas as scrotal swellings have been reported. The gold standard treatment for such tumors is radical orchidectomy followed by a multimodal approach including radiotherapy and chemotherapy [8,9].

In the present case, the patient had swelling in the para-scrotal region over 15 years and it started growing progressively leading to swelling bigger than the scrotum. On investigations, we suspected malignant conversion of the lipoma to liposarcoma which was confirmed postoperatively with histopathological report. A high index of suspicion is essential for such conversion of benign lipomatous swelling to liposarcoma. Patients with multiple lipomatoses should be primed for the possibility of such complications. Wide local excision is a must and can be followed by adjuvant chemoradiation with bad prognostic markers mentioned above.

## Conclusions

A high index of suspicion is essential to diagnose liposarcomas as they present clinically as benign growths. The decision to perform a radical surgery should be considered when there is a clinico-radiological suspicion of a neoplastic growth as the pre-operative biopsy may be misleading. The treatment for low-grade tumors is wide local excision alone. Adjuvant radiotherapy for large tumors (>10 cm) has been known to increase the disease-free survival period. Regular follow up required to diagnose recurrence at the earliest.
